# Screening performance of the MUS-3600 urine analyzer for urinary tract infection: comparison with urine culture

**DOI:** 10.3389/abp.2026.16555

**Published:** 2026-07-16

**Authors:** Zeynep Mine Yalçınkaya Kara, Leyla Genç, Merve Sena Odabaşı, Elif Aktaş

**Affiliations:** 1 Department of Biochemistry, University of Tekirdağ Namık Kemal, Tekirdağ, Türkiye; 2 Department of Microbiology, University of Health Sciences Şişli Hamidiye Etfal Training and Research Hospital, İstanbul, Türkiye; 3 Department of Biochemistry, University of Health Sciences Şişli Hamidiye Etfal Training and Research Hospital, İstanbul, Türkiye

**Keywords:** automated urinalysis, flow-type micro-imaging technology, MUS-3600, urine culture, UTI

## Abstract

**Background:**

Urine culture is the gold standard for diagnosing urinary tract infections (UTIs), but most samples yield negative results. A rapid and accurate screening method could accelerate reporting, reduce workload, and lower costs. This study aimed to evaluate the diagnostic performance of the MUS-3600 urine analyzer as a screening tool for UTIs.

**Methods:**

Urine samples submitted to the microbiology laboratory were analyzed using the MUS-3600 within 10 min of collection. The analyzer uses flow-type micro-imaging and artificial intelligence–based particle classification. Culture results with ≥10^4^ CFU/mL were defined as positive; lower counts were negative.

**Results:**

For white blood cells (WBC, >39/μL), sensitivity, specificity, PPV, NPV, and elimination rate were 63.4%, 84.5%, 50.8%, 90.1%, and 67.5%, respectively. Bacilli (>103/μL) showed 62.1%, 88.1%, 56.9%, 90.2%, and 70.4%. Suspected cocci (>859/μL) demonstrated lower accuracy (44.4%, 78.7%, 34.5%, 84.9%, and 62.8%). Combining WBC and bacilli yielded the highest elimination rate (77.2%) and NPV (87.9%). The analyzer performed better in males (NPV 93.9%) than in females (NPV 82.1%).

**Conclusion:**

The MUS-3600 provides moderate accuracy for UTI screening and may help reduce unnecessary urine cultures, particularly in male patients. However, its limited sensitivity, especially for cocci, prevents its use as a stand-alone screening tool. Further studies are needed to confirm its role as a complementary method for culture elimination.

## Introduction

Urinary tract infections (UTIs) are among the most common community-acquired and hospital-acquired infections worldwide. Urine culture is considered the gold standard for laboratory diagnosis of UTI (Del Ben et al., 2023; Monsen and Ryden, 2017; Herráez et al., 2018; Erdman et al., 2017). However, the culture process typically requires 24–48 h and represents a significant workload for microbiology laboratories. In clinical laboratories, more than half of the samples sent for urine culture are negative (Del Ben et al., 2023; Monsen and Ryden, 2017). Many patients are also started on empirical antibiotics before a diagnosis is made, contributing to the development of antibiotic resistance (Del Ben et al., 2023; Monsen and Ryden, 2017; Herráez et al., 2018; Erdman et al., 2017; Kim et al., 2019).

The development of screening methods with high sensitivity and negative predictive value (NPV) to identify negative samples before culture is essential. A rapid and reliable screening method would shorten the time required to report negative results, reduce the laboratory workload and costs, and facilitate earlier clinical decision-making, including patient discharge. It would also reduce unnecessary empirical antibiotic use and, consequently, the development of antibiotic resistance (Del Ben et al., 2023; Monsen and Ryden, 2017; Herráez et al., 2018; Erdman et al., 2017; Ilki et al., 2017; Wang et al., 2023; Millán-Lou et al., 2018).

Urinalysis is essential for diagnosing UTIs, and recent advances in automated analysis technologies, particularly flow cytometry-based systems, have enabled comparisons with conventional culture methods. Most studies have mainly focused on evaluating leukocytes and bacteria together (Del Ben et al., 2023; Monsen and Ryden, 2017; Chun et al., 2022).

In addition to conventional parameters, emerging urine analyzers have introduced novel particle classification capabilities, including differentiation between bacterial morphotypes and other formed elements. These advances may offer incremental diagnostic value beyond traditional leukocyte and total bacteria counts, particularly in distinguishing true infection from contamination. However, the clinical utility and added value of these extended parameters remain insufficiently validated in routine practice. Therefore, further evidence is needed to clarify whether these advanced analytical outputs can meaningfully improve screening performance and optimize culture utilization in real-world laboratory settings.

In this study, midstream urine samples sent to the microbiology laboratory for culture were simultaneously analyzed using the MUS-3600 (Dirui Industrial Changchun, China). The diagnostic performance of the analyzer was evaluated by comparing its results with those of urine culture as the reference standard.

## Materials and methods

### Collection of urine specimens and analyzers

This study was conducted in a tertiary hospital between 15 February 2024 and 28 February 2024. The samples were collected in Sarstedt V-Monovette urine tubes (SARSTEDT AG & Co.KG. Nümbrecht, GERMANY). Urine samples for culture and urine analysis were collected and transported to the laboratory without delay. The samples were collected in a sterile culture containers with screw caps. All samples that met the inclusion criteria were accepted.

Urine culture and urinalysis were performed simultaneously on a total of 856 midstream urine specimens collected from outpatient clinics and intensive care units within 1 h of arrival at the laboratory. The age range of the patients included in the study was 18–96 years. Patients under 18 years of age, those with urinary catheter specimens, samples with insufficient volume, or specimens with excessive hematuria and mucus during visual inspection were excluded. Except for intensive care unit patients, no antibiotic use was reported in outpatient clinics or inpatients prior to culture.

All samples were cultured for quantitative urine culture without delay and then analyzed within 10 min using the MUS-3600 fully automatic urine analyzer.

MUS-3600 is a system that integrates the H1600 Urine Analyzer and the FUS360 Urine Sediment Analyzer using flow-type micro-imaging technology. Cells and other parameters are identified and classified using artificial intelligence-based identification (AII).

### Urine culture

For culture, urine samples were plated on chromogenic agar (CPSE/Biomerieux, France) and incubated at 35 °C–37 °C for 18–24 h. Culture results were evaluated as positive if the colony count was ≥10^4^ cfu/mL for a single pathogen or each of two potential pathogens, and as negative if < 10^4^ cfu/mL (Haugum et al., 2021). Cultures showing three or more species without a dominant organism were considered contaminated by urethral flora and excluded. Microorganisms were identified using Matrix-Assisted Laser Desorption/Ionization- Time of Flight (MALDI-TOF) mass spectrometry (Biomerieux, France), and antibiotic susceptibility testing was performed using the VITEK 2 Compact (Biomerieux, France).

Of the 856 patients, 97 were considered to have contaminated results according to the culture and were excluded from the evaluation. Of the 759 evaluated samples, 409 (53.9%) were from female patients, and 350 (46.1%) were from male patients. A total of 729 (96.0%) samples were from outpatients, 26 (3.5%) from inpatients, and 4 (0.5%) from intensive care unit patients. Based on the parameters in Table 1, culture positive and negative samples were compared.

**TABLE 1:** Distribution of demographic and clinical findings according to culture growth.

Characteristics	Total (n = 759)	Culture (−) (n = 606)	Culture (+) (n = 153)	p-value
Male, n (%)	350 (46.1)	308 (50.8)	42 (27.5)	​
Female, n (%)	409 (53.9)	298 (49.2)	111 (72.5)	<0.001
Age (years)	55 (19–96)	54 (19–96)	58 (20–96)	0.001
WBC (/µL)	7 (0–22779)	5 (0–22779)	105 (0–17992)	<0.001
WBC cluster (/µL)	0 (0–1,316)	0 (0–1,316)	0 (0–1,021)	<0.001
Squamous epithelial cell (/µL)	3 (0–502)	2.5 (0–479)	8 (0–502)	<0.001
Non-squamous epithelial cell (/µL)	0 (0–41)	0 (0–38)	0 (0–41)	<0.001
Transitional epithelial cell (/µL)	0 (0–41)	0 (0–38)	0 (0–41)	<0.001
Hyaline cast (/µL)	0 (0–48)	0 (0–48)	0 (0–9)	0.524
Granular cast (/µL)	0 (0–6)	0 (0–6)	0 (0–4)	0.030
*Bacillus* (/µL)	0 (0–40483)	0 (0–3,515)	294 (0–40483)	<0.001
Suspected coccus (/µL)	374 (0–40404)	328 (0–17587)	650 (0–40404)	<0.001
Total bacteria (/µL)	408 (0–59435)	337.5 (0–17587)	1,080 (0–59435)	<0.001
Yeast (/µL)	0 (0–1716)	0 (0–121)	0 (0–1716)	0.001
Crystal (/µL)	0 (0–965)	0 (0–965)	0 (0–871)	0.230
Calcium oxalate (/µL)	0 (0–687)	0 (0–684)	0 (0–687)	0.444
Mucous strands (/µL)	24 (0–1,277)	26 (0–1,277)	13 (0–851)	0.006
pH	6 (5.50–8)	6 (5.50–8)	6 (5.50–8)	0.747
Nitrite negative, n (%)	718 (94.6)	604 (99.7)	114 (74.5)	​
Nitrite positive, n (%)	41 (5.4)	2 (0.3)	39 (25.5)	<0.001
Leukocyte negative, n (%)	545 (71.8)	491 (81)	54 (35.3)	​
Leukocyte positive, n (%)	214 (28.2)	115 (19)	99 (64.7)	<0.001

Data are presented as median (min–max) for continuous variables and number (percentage) for categorical variables. Mann–Whitney U test and Chi-square/Fisher’s exact tests were used as appropriate.

### Statistical methods

Patient data were analyzed using IBM Statistical Package for the Social Sciences (SPSS) for MacOS version 29.0 (IBM Corp, Armonk, NY). For categorical variables, frequencies and percentages were reported, while for continuous variables, mean, standard deviation, median, minimum, and maximum values were provided. Normality tests were performed using the Kolmogorov-Smirnov test. The Mann-Whitney U test was used for non-parametric data, and the Chi-square or Fisher’s exact test for categorical variables. Receiver Operating Characteristic (ROC) analysis was performed for parameters expected to have a distinguishing effect on culture growth, and the area under the curve (AUC) was calculated. The most appropriate cut-off values for leukocyte and bacterial screening were determined. Sensitivity, specificity, PPV, and NPV were calculated by referencing the culture results as the gold standard method. Spearman’s correlation analysis was used to examine relationships between continuous variables. Results with a p-value of less than 0.05 were considered statistically significant.

Optimal cut-off values were determined using the Youden index (J) according to the presence of growth in culture. The elimination rate was calculated as the ratio of true negative samples to all tested samples (TN/total × 100).

## Results

When culture results were evaluated in this study, 153 (20.2%) of 759 samples showed growth of specific uropathogens, while 606 (79.8%) showed no growth. Positive samples were predominantly from female patients (111, 72.5%).

Table 1 shows the distribution of demographic and clinical findings based on the presence or absence of culture growth. A statistically significant difference was observed between the groups for all demographic and clinical variables except hyaline cast, crystal, calcium oxalate, and pH (p > 0.05). Among the 41 nitrite-positive samples, 39 showed growth in culture.

When examining the distribution of microorganisms, *Escherichia coli* was the most dominant uropathogen, accounting for 44.4% of the cases. It was followed by *Streptococcus agalactiae* (15.7%), *Enterococcus faecalis* (14.4%), and *Klebsiella pneumoniae* (11.8%) (Table 2).

**TABLE 2:** Distribution of microorganisms Grown in patients with culture growth*.

Classification	Microorganisms	n (%)
Bacilli	*E.coli*	68 (44.4)
​	*K.pneumoniae*	18 (11.8)
​	*P. aeruginosa*	7 (4.6)
​	*P. mirabilis*	6 (3.9)
​	*K. aerogenes*	4 (2.6)
​	*E. cloacae*	3 (2)
​	Other	7 (4.6)
Cocci	*S. agalactiae*	24 (15.7)
​	*E. faecalis*	22 (14.4)
​	*S. aureus*	5 (3.3)
​	*S. epidermidis*	3 (2)
​	Other	6 (3.9)
Yeast	*C. tropicalis*	2 (1.3)
​	*C. albicans*	1 (0.7)
Other	​	8 (5.2)

* Multiple microorganisms were isolated in some samples. Data are presented as number (percentage).

Table 3 presents the results of correlation analyses evaluating the relationship between WBC, bacilli count and other demographic and clinical variables. WBC showed a very weak but statistically significant correlation with hyaline cast, granular cast, yeast, crystal, mucus; a weak significant correlation with nitrite and suspected cocci; a moderate significant correlation with WBC cluster, squamous epithelial cell, non-squamous epithelial cells, transitional epithelium and total bacterial count; and a strong positive significant correlation with strip leukocytes.

**TABLE 3:** Relationship between WBC and *bacillus* count and other parameters.

Parameter	WBC (r, p)	*Bacillus* (r, p)
White blood cell cluster	0.477, <0.001	0.331, <0.001
Squamous epithelial cell	0.381, <0.001	0.465, <0.001
Non-squamous epithelial cell	0.408, <0.001	0.227, <0.001
Transitional epithelial cell	0.408, <0.001	0.227, <0.001
Hyaline cast	0.086, 0.018	0.038, 0.294
Granular cast	0.091, 0.012	0.045, 0.217
Suspected coccus	0.356, <0.001	0.424, <0.001
Total bacteria number	0.395, <0.001	0.568, <0.001
Yeast	0.090, 0.014	0.132, <0.001
Crystal	0.103, 0.005	−0.016, 0.655
Calcium oxalate	0.069, 0.057	−0.046, 0.206
Mucous strands	0.135, <0.001	0.025, 0.486
pH	−0.049, 0.180	0.103, 0.005
Age	0.003, 0.936	−0.118, 0.001
Nitrite	0.295, <0.001	0.418, <0.001
Leukocytes	0.671, <0.001	0.376, <0.001

Spearman’s rank correlation analysis was used to assess relationships between continuous variables. Values are presented as correlation coefficient (r) and p-value.

*Bacillus* showed a very weak positive significant correlation with yeast, pH; a weak with non-squamous epithelial cell, WBC cluster, leukocyte, transitional epithelial cell, moderate positive significant correlation with squamous epithelial cell, suspected cocci, nitrite, and total bacterial count. A very weak negative correlation with age was also observed.

**FIGURE 1:**
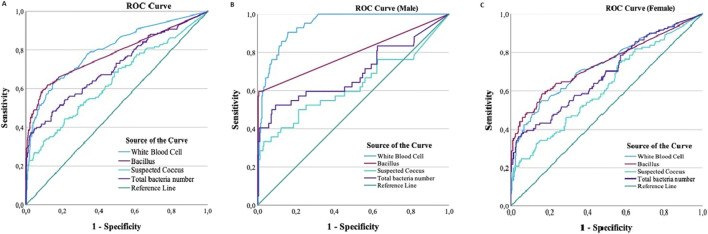
Receiver operating characteristic (ROC) curves of MUS-3600 parameters for the prediction of urine culture positivity in the overall population **(A)**, male patients **(B)**, and female patients **(C)**.

The ROC curves for WBC, *bacillus*, suspected cocci, and total bacterial count, based on culture growth in male and female patients, are shown in Figure 1.

The diagnostic performance metrics at different cut-off points, along with gender-specific analyses, are presented in Table 4. The area under the curve (AUC) for WBC was 0.798, and the cut-off value was determined to be 39, with a sensitivity of 63.4%, specificity of 84.5%, PPV of 50.8%, NPV of 90.1%, and an elimination rate of 67.5%. For bacilli and suspected cocci, the AUC values were 0.778 and 0.642, respectively, with cutoff values of 103 and 859. The sensitivity, specificity, PPV, NPV, and elimination rate for bacilli were 62.1%, 88.1%, 56.9%, 90.2%, and 70.4%, respectively, while for suspected cocci, these values were 44.4%, 78.7%, 34.5%, 84.9%, and 62.8%, respectively.

**TABLE 4:** Diagnostic performance of MUS-3600 parameters for UTI screening.

Group	Risk factor	AUC (95% CI)	Cut-off	Sensitivity (%)	Specificity (%)	TP	FN	TN	FP	PPV (%)	NPV (%)	Elimination rate (%)
ALL (n = 759)	WBC (/μL)	0.798 (0.755–0.841)	>39	63.4	84.5	97	56	512	94	50.8	90.1	67.5
	Bacillus (/μL)	0.778 (0.729–0.828)	>103	62.1	88.1	95	58	534	72	56.9	90.2	70.4
	Suspected coccus (/μL)	0.642 (0.589–0.696)	>859	44.4	78.7	68	85	477	129	34.5	84.9	62.8
	Total bacteria (/μL)	0.710 (0.660–0.761)	>2,839	39.2	95.5	60	93	579	27	69.0	86.2	76.3
	WBC + Bacillus*	0.84 (0.78–0.88)	—	47.1	96.7	72	81	586	20	78.3	87.9	77.2
MALE (n = 350)	WBC (/μL)	0.941 (0.913–0.969)	>39	90.5	84.1	40	4	257	49	43.7	98.5	73.4
	Bacillus (/μL)	0.793 (0.698–0.887)	>73	57.1	99.4	25	19	304	2	92.3	94.4	86.9
	Suspected coccus (/μL)	0.624 (0.513–0.734)	>2,382	40.5	86.0	18	26	263	43	28.3	91.4	75.1
	Total bacteria (/μL)	0.692 (0.586–0.797)	>1,139	40.5	97.4	18	26	298	8	68.0	92.3	85.1
	WBC + Bacillus*	0.91 (0.85–0.95)	—	52.4	99.4	23	21	304	2	91.7	93.9	86.9
FEMALE (n = 409)	WBC (/μL)	0.732 (0.673–0.791)	>34	53.2	84.9	58	51	255	45	56.7	83.0	62.3
	Bacillus (/μL)	0.740 (0.679–0.801)	>158	64.0	76.5	70	39	230	70	50.4	85.1	56.2
	Suspected coccus (/μL)	0.621 (0.558–0.684)	>1,643	45.9	71.1	50	59	213	87	37.2	77.9	52.1
	Total bacteria (/μL)	0.687 (0.626–0.748)	>2,816	38.7	93.6	42	67	281	19	69.4	80.4	68.7
	WBC + Bacillus*	0.78 (0.71–0.81)	—	45.0	94.0	49	60	282	18	73.5	82.1	68.9

Receiver operating characteristic (ROC) analysis was performed to evaluate diagnostic performance. The area under the curve (AUC) with 95% confidence intervals (CI) was calculated. AUC, Area Under the Curve; CI, Confidence Interval; NPV, Negative Predictive Value; PPV, Positive Predictive Value; WBC, White Blood Cells, TP, True Positive, FN, False Negative, TN, True Negative, FP, False Positive.

* Both WBC and *Bacillus* parameters must be positive (WBC >39/μL AND *Bacillus* >103/μL).

When WBC (>39) and *bacillus* (>103) were evaluated together as a screening method (both parameters positive), the highest overall specificity (96.7%), PPV (78.3%), NPV (87.9%), and elimination rate (77.2%) were observed, although sensitivity decreased to 47.1%. In subgroup analysis, this combination gave an elimination rate of 86.9% (NPV: 93.9%) in males and 68.9% (NPV: 82.1%) in females. The performance was notably better in male patients, with WBC alone showing a sensitivity of 90.5% and NPV of 98.5% at the same cut-off.

## Discussion

Urine samples constitute a substantial proportion of laboratory workload, and a large proportion are reported as either culture-negative or contaminated. In this context, rapid and reliable screening strategies are essential to reduce unnecessary culture requests and inappropriate empirical antimicrobial use (Ilki et al., 2017; Dong et al., 2022; Íñigo et al., 2016; De Rosa et al., 2018).

In the present study, we evaluated the performance of the MUS-3600 urine analyzer as a screening tool by comparing its parameters with urine culture results. The strong concordance observed between nitrite positivity and culture growth supports the established role of nitrite as a reliable rule-in indicator in routine practice. In addition, the predominance of *Escherichia coli* among uropathogens is consistent with well-established epidemiological patterns reported in previous studies (Monsen and Ryden, 2017; Erdman et al., 2017; Kim et al., 2019).

Among the evaluated parameters, WBC demonstrated moderate diagnostic accuracy with a relatively high negative predictive value, supporting its utility as a screening marker for excluding infection. Feng Dong et al. reported a lower NPV for WBC (75.3%), whereas the higher NPV observed in our study may be attributable to differences in sample size and study design (Dong et al., 2022).

*Bacillus* showed a comparable level of diagnostic performance to WBC, whereas suspected coccus exhibited clearly inferior performance, indicating limited clinical utility when used alone. In the study by Dong et al., the optimal cut-off value for bacilli was reported as 50, with a sensitivity of 69.5% and an NPV of 82%. In contrast, we identified a higher optimal cut-off value for bacilli, while maintaining a broadly comparable diagnostic profile, suggesting potential variability related to device-specific characteristics and population differences (Dong et al., 2022).

When the overall screening performance was evaluated, WBC demonstrated slightly higher discriminative ability compared to *bacillus*, total bacterial count, and suspected coccus. In contrast, Wang et al. reported superior performance for bacterial parameters (AUC: 0.86) compared to WBC (AUC: 0.74) using a different analytical system, and suggested that combining leukocyte and bacterial parameters could improve diagnostic performance (Wang et al., 2023). Consistent with this observation, our findings indicate that combining WBC and *bacillus* parameters enhances screening performance.

Millan Lou et al., in a comparative study of two different systems, reported that bacterial counts demonstrated higher diagnostic performance than leukocytes, and that combining bacterial and leukocyte parameters improved overall accuracy (Millán-Lou et al., 2018). In line with these findings, the combination of WBC and *bacillus* in our study resulted in improved specificity and negative predictive value, albeit with a reduction in sensitivity, reflecting a trade-off inherent to screening strategies.

Similarly, Koçer et al. reported comparable diagnostic performance between bacterial and leukocyte parameters (AUC: 0.710 and 0.728, respectively), which is consistent with the similar performance observed in our study (Kocer et al., 2014). In contrast, Haugum et al. reported substantially higher AUC values for both bacterial and leukocyte counts (0.94 and 0.81, respectively), highlighting variability across different analytical platforms and study populations (Haugum et al., 2021).

The combined use of WBC and *bacillus* provided the highest overall screening efficiency in our study. Notably, subgroup analysis revealed superior performance in male patients compared to females. This difference may be explained by higher contamination rates in female urine samples due to anatomical factors, leading to reduced specificity and screening efficiency. Similar sex-related differences in screening performance have been reported in previous studies (Ilki et al., 2017; Dong et al., 2022; Kouri et al., 2024). These findings suggest that the use of gender-specific cut-off values may improve diagnostic accuracy in routine practice.

In male patients, *bacillus* demonstrated particularly high specificity and positive predictive value at optimized cut-off levels, indicating that this parameter may be especially useful for ruling in infection and guiding early antimicrobial therapy decisions when Gram-negative UTI is suspected. In contrast, suspected coccus showed limited diagnostic value, with relatively low predictive performance. Hua Wang et al. similarly reported that Gram-negative flags demonstrated high positive predictive value, whereas Gram-positive indicators were less reliable, further supporting the limited clinical utility of suspected coccus as an isolated parameter (Wang et al., 2023).

From a clinical perspective, an effective screening strategy for UTI should primarily aim to achieve high sensitivity and negative predictive value in order to safely exclude infection. According to European Urinalysis Guidelines, screening methods for clinically significant bacteriuria are expected to achieve sensitivity levels of 90%–95% (Kouri et al., 2024). In our study, although high negative predictive values were achieved, sensitivity levels remained below the recommended range in the overall population, with the exception of male patients, indicating that while the system is effective for ruling out infection, caution is required when using it as a standalone diagnostic tool.

### Clinical implications and limitations

From a clinical perspective, although a 100% negative predictive value was not achieved, the consistently high NPV, particularly among male patients, suggests that the system may be used for preliminary risk stratification in routine laboratory workflows. A secondary objective of this study was to minimize unnecessary empirical or prophylactic antibiotic use while ensuring that no clinically significant UTI cases were overlooked. However, relying solely on automated parameters carries an inherent risk of missing infections; therefore, urine culture must remain the reference standard for definitive diagnosis.

This study was conducted at a single center and the cut-off values were derived from our study population. However, the relatively large sample size (n = 759) enhances the robustness of the findings, and the analyses, reflecting routine clinical laboratory practice, support their practical applicability. Diagnostic performance may vary according to patient demographics, prior antibiotic exposure, inpatient versus outpatient distribution, and local microbiological epidemiology (Daldaban Dinçer et al., 2019). For this reason, laboratories should validate and optimize their own cut-off values in accordance with local conditions before routine implementation.

## Conclusion

The MUS-3600 analyzer generally demonstrated moderate diagnostic performance. In the study cohort, the highest overall elimination rate (77.2%) and negative predictive value (87.9%) were obtained when WBC (>39/μL) and bacilli (>103/μL) were evaluated together. These results suggest that unnecessary cultures can be reduced in certain cases. However, the relatively low sensitivity (44.4%) and positive predictive value (34.5%) observed in the classification of suspicious cultures limit the reliability of this parameter when used independently.

Subgroup analysis showed better performance in male patients with an elimination rate of 86.9% and an NPV of 93.9% compared to female patients with an elimination rate of 68.9% (NPV: 82.1%). This gender-related variability indicates that diagnostic efficiency can be improved by using specific cut-off strategies. While these findings support the use of MUS-3600 as a screening tool, none of the evaluated parameters reached the 100% NPV required for the safe exclusion of infection. In absolute terms, this corresponds to approximately 20 missed infections among 350 male patients (6%) and 61 cases among 409 female patients (15%) in our cohort. Therefore, culture decisions should be individualized, especially for high-risk populations such as immunocompromised individuals and intensive care unit patients.

In conclusion, flow-type microimaging systems such as MUS-3600 provide a valuable contribution to laboratory workflows. They are not intended to replace urine culture but rather to complement it by improving laboratory workflow efficiency. These findings need to be further validated in larger prospective studies, and it is necessary to investigate whether gender-specific cut-off values or advanced analytical approaches, including machine learning, can improve diagnostic performance.

## Data Availability

The raw data supporting the conclusions of this article will be made available by the authors, without undue reservation.
